# Quinine Effects on Gut and Pancreatic Hormones and Antropyloroduodenal Pressures in Humans–Role of Delivery Site and Sex

**DOI:** 10.1210/clinem/dgac182

**Published:** 2022-03-24

**Authors:** Peyman Rezaie, Vida Bitarafan, Braden D Rose, Kylie Lange, Jens F Rehfeld, Michael Horowitz, Christine Feinle-Bisset

**Affiliations:** Adelaide Medical School and Centre of Research Excellence in Translating Nutritional Science to Good Health, University of Adelaide, Adelaide SA 5005, Australia; Adelaide Medical School and Centre of Research Excellence in Translating Nutritional Science to Good Health, University of Adelaide, Adelaide SA 5005, Australia; Adelaide Medical School and Centre of Research Excellence in Translating Nutritional Science to Good Health, University of Adelaide, Adelaide SA 5005, Australia; Adelaide Medical School and Centre of Research Excellence in Translating Nutritional Science to Good Health, University of Adelaide, Adelaide SA 5005, Australia; Department of Clinical Biochemistry, Rigshospitalet, 2100 Copenhagen, Denmark; Adelaide Medical School and Centre of Research Excellence in Translating Nutritional Science to Good Health, University of Adelaide, Adelaide SA 5005, Australia; Endocrine and Metabolic Unit, Royal Adelaide Hospital, Adelaide SA 5005, Australia; Adelaide Medical School and Centre of Research Excellence in Translating Nutritional Science to Good Health, University of Adelaide, Adelaide SA 5005, Australia

**Keywords:** gut functions, bitter taste, appetite-regulatory hormones, glucoregulatory hormones, gut motility, human

## Abstract

**Context:**

The bitter substance quinine modulates the release of a number of gut and gluco-regulatory hormones and upper gut motility. As the density of bitter receptors may be higher in the duodenum than the stomach, direct delivery to the duodenum may be more potent in stimulating these functions. The gastrointestinal responses to bitter compounds may also be modified by sex.

**Background:**

We have characterized the effects of intragastric (IG) versus intraduodenal (ID) administration of quinine hydrochloride (QHCl) on gut and pancreatic hormones and antropyloroduodenal pressures in healthy men and women.

**Methods:**

14 men (26 ± 2 years, BMI: 22.2 ± 0.5 kg/m^2^) and 14 women (28 ± 2 years, BMI: 22.5 ± 0.5 kg/m^2^) received 600 mg QHCl on 2 separate occasions, IG or ID as a 10-mL bolus, in randomized, double-blind fashion. Plasma ghrelin, cholecystokinin, peptide YY, glucagon-like peptide-1 (GLP-1), insulin, glucagon, and glucose concentrations and antropyloroduodenal pressures were measured at baseline and for 120 minutes following QHCl.

**Results:**

Suppression of ghrelin (*P* = 0.006), stimulation of cholecystokinin (*P* = 0.030), peptide YY (*P* = 0.017), GLP-1 (*P* = 0.034), insulin (*P* = 0.024), glucagon (*P* = 0.030), and pyloric pressures (*P* = 0.050), and lowering of glucose (*P* = 0.001) were greater after ID-QHCl than IG-QHCl. Insulin stimulation (*P* = 0.021) and glucose reduction (*P* = 0.001) were greater in females than males, while no sex-associated effects were found for cholecystokinin, peptide YY, GLP-1, glucagon, or pyloric pressures.

**Conclusion:**

ID quinine has greater effects on plasma gut and pancreatic hormones and pyloric pressures than IG quinine in healthy subjects, consistent with the concept that stimulation of small intestinal bitter receptors is critical to these responses. Both insulin stimulation and glucose lowering were sex-dependent.

There is increasing interest in the gastrointestinal (GI) effects of bitter substances, stimulated by preclinical observations that a number of bitter compounds have potent effects to modulate gut and glucoregulatory hormones (e.g., cholecystokinin [CCK], glucagon-like peptide [GLP-1]) as well as upper GI motility, which underlies the slowing of gastric meal emptying (1-4). This is of particular interest, because, as both GI hormones and gastric emptying contribute to the regulation of energy intake and postprandial blood glucose (5), there are substantial implications for the management of obesity and type 2 diabetes.

Bitter substances are sensed in the GI lumen by bitter taste receptors, located on enteroendocrine cells (6). In both cell lines and experimental animals, a number of bitter agonists have been shown to potently stimulate gut hormones, particularly ghrelin, CCK, and GLP-1 (3, 7-9). Some bitter substances have also been found to slow gastric emptying (1), possibly reflecting direct effects on smooth muscle contractility (1). These preclinical observations have attracted recent interest to establish whether they can be replicated in humans and, if so, whether they are associated with reductions in energy intake and/or blood glucose (5). Quinine is probably the best-studied bitter substance in humans. It is used clinically to treat malaria, usually in a dose of 500 mg intravenously (10), and has the capacity to induce hypoglycemia, probably by direct stimulation of insulin (11). A small number of studies have reported effects of quinine on the secretion of gut hormones, including suppression of plasma ghrelin, and stimulation of CCK and GLP-1 (12-16). Intragastric (IG) administration of quinine hydrochloride (QHCl), in a dose of 10 µmol/kg (equivalent to ~270 mg in a 70-kg human), suppressed ghrelin (14, 15), while a recent study from the same group found no effect of either intraduodenal (ID) or IG-QHCl, in the same dose, on plasma CCK (17). In contrast, an IG bolus of 18 mg QHCl was found to increase plasma CCK (although the effect was very small) after a standardized meal (12). Both ID- and IG-QHCl, in a dose of 600 mg (~500 mg quinine), stimulated GLP-1 and insulin, associated with postprandial blood glucose lowering (13, 16). The effect of quinine on peptide YY (PYY) is not known.

Animal studies indicate that the density of bitter taste receptors is greater in the duodenum than the stomach (6, 18), suggesting that ID administration may have more potent effects than IG delivery to modulate GI hormones and motility. Indeed, in our recent studies, QHCl, in a dose of 600 mg, administered either ID 30 minutes before, or IG 60 minutes (to allow time for small intestinal exposure) before, a mixed-nutrient drink, had comparable effects to slow gastric emptying (16), while IG administration 30 minutes before the drink had no effect (13). The comparative effects of ID and IG quinine on the release of gut (ie, ghrelin, CCK, PYY, GLP-1) and pancreatic (ie, insulin, glucagon) hormones and upper GI motility in humans have not been evaluated.

Oral sensitivity to bitter substances may be higher in women than men, as reflected in lower detection thresholds for the bitter substance, 6-n-propylthiouracil (19). That their response to GI exposure may also be enhanced, is supported by a report that IG administration of denatonium benzoate affected the origin of phase III of fasting motility and decreased hunger and energy intake in women, but not men (20). Whether there are sex differences in the effects of bitter substances on gut and pancreatic hormone secretion, or the changes in upper GI motility that underlie the slowing of gastric emptying, is not known.

The aims of this study were to determine the effects of the site of administration, that is, stomach or duodenum, of the bitter substance QHCl on gut and pancreatic hormones and antropyloroduodenal pressures in healthy individuals and to evaluate whether any effects are influenced by sex.

## Methods

### Study Participants

14 healthy males (mean age: 26 ± 2 years, body mass index [BMI]: 22.2 ± 0.5 kg/m^2^) and 14 healthy premenopausal females (mean age: 28 ± 1.9 years, BMI: 22.5 ± 0.5 kg/m^2^) participated in the study. Because of a lack of suitable prior data, we were unable to perform power calculations. However, based on our recent studies, we calculated that n = 14 participants would allow detection of a difference of 5 pmol/L, with an SD of 6 pmol/L, for plasma GLP-1 (16), and a difference of 15, with a SD of 14, for the number of pyloric pressure waves (21), at α = 0.05 and a power of 80%. Participants were recruited through flyers placed around local universities and the Royal Adelaide Hospital, and advertisements in local newspapers. Each was screened before their inclusion to exclude those with GI symptoms or a history of GI disease or surgery; vegetarians; smokers; overweight or obese individuals (BMI > 25 kg/m^2^); an alcohol consumption of > 20 g/day on > 5 days/week; use of medications known to affect appetite, energy intake, or GI function; high-performance athletes; unstable body weight (≥5% change over the last 3 months before participation); and restrained eaters (score > 12 on the restrained eating component of the 3-factor eating questionnaire) (22). Oral taste detection thresholds for QHCl were quantified, using the ascending-series 3-alternative forced-choice technique (13, 23), to ensure that all participants detected QHCl. The study protocol was approved by The Human Research Ethics Committee of the Central Adelaide Local Health Network and performed in accordance with the Declaration of Helsinki. All participants provided written, informed consent before their inclusion, and after enrollment each was assigned to a treatment order of balanced randomization that was generated with an online tool (www.randomization.com) by a research officer who was not involved in data analysis. The study was registered as a clinical trial with the Australian and New Zealand Clinical Trials Registry (www.anzctr.org.au; ACTRN12619000707167) and performed from May 2019 to August 2020.

### Study Outline

The study evaluated the effects of IG and ID administration of QHCl, in a dose of 600 mg, on gut (ie, ghrelin, CCK, PYY, GLP-1) and pancreatic (ie, insulin and glucagon) hormones and antropyloroduodenal pressures in healthy lean men and women. Plasma glucose was also measured, primarily to ensure that concentrations remained within physiological levels. Hormone concentrations and antropyloroduodenal pressures were primary, while plasma glucose and GI symptoms were secondary, outcomes. We employed intragastric and intraduodenal administration to ensure standardized and targeted delivery of QHCl to the desired site.

### Preparation of QHCl Solution

The QHCl solution was prepared by dissolving 600 mg QHCl (Sinkona Indonesia Lestari, Subang, West Java, Indonesia) in 10 mL distilled water. The solution was prepared on the morning of each study day, and filled in a syringe, by a research officer who had no involvement in data analysis, and administered at a temperature of ~30 °C. The dose of QHCl was chosen based on our previous study, in which ID and IG administration of 600 mg QHCl were shown to stimulate GLP-1, slow gastric emptying, and reduce postprandial blood glucose (16).

### Study Protocol

Each participant was studied on 2 occasions, separated by 3 to 7 days, in randomized, double-blind fashion. Studies in women were performed during the follicular phase of their menstrual cycle (ie, days 1-8) to minimize a potential confounding effect (24). Participants were instructed to refrain from vigorous physical activity and alcohol consumption for 24 hours prior to each study and, were provided with a standardized meal (beef lasagna; total energy content: 602 kcal; McCain Food, Wendouree, Victoria, Australia) to be consumed between 6:30 and 7 pm on the evening prior to each study visit. The following morning, each participant attended the Clinical Research Facility at the Adelaide Medical School, University of Adelaide, at 8:15 am after an overnight fast (from both solids and liquids, with the exception of water, after 7 pm, and from water after 6.30 am).

Upon arrival, each participant was intubated with a manometric catheter (Dentsleeve International, Mui Scientific, Mississauga, Ontario, Canada; total length: 100 cm; external diameter: 3.5 mm), which was inserted through an anesthetized nostril into the stomach and allowed to pass into the duodenum by peristalsis. The catheter included 6 channels positioned in the antrum, a 4.5-cm pyloric sleeve sensor with 2 channels situated on its back, and 7 channels positioned in the duodenum, with all side-holes spaced at 1.5-cm intervals, measuring pressures in the antrum, pylorus, and duodenum (25). The correct positioning of the catheter, with the sleeve sensor straddling the pylorus, was maintained by continuous measurement of the transmucosal potential difference between the most distal antral, and most proximal duodenal, channels (26). The most proximal antral channel was used for IG administration of quinine, and an additional infusion channel, located ~14.5 cm distal to the pylorus, for ID administration.

Once the catheter was in position (within 55 ± 10 min), an intravenous cannula was placed into a forearm vein, and the arm was kept warm with a heat pad for regular sampling of “arterialized” blood. Following the occurrence of phase III of fasting motility (a distinct motor pattern characterized by high-frequency, high-amplitude contractions designed to clear the GI lumen of food remnants), during phase I (a period of motor quiescence), fasting motility was monitored for 10 minutes (t = −10 to 0 minutes), and at t = −1 minute, QHCl was administered, within 1 minute, into either the stomach or duodenum. Blood samples were taken at baseline (t = −10 minutes) and following quinine administration, at t = 0, 10, 20, 30, 45, 60, 75, 90, and 120 minutes, for measurement of plasma concentrations of ghrelin, CCK, PYY, GLP-1, insulin, glucagon, and glucose. Visual analog scale (VAS) ratings, to assess GI symptoms, ie, bloating and nausea, were collected at the same time points. Antropyloroduodenal pressures were measured continuously for 120 minutes. At t = 120 minutes, the catheter and the intravenous cannula were removed, each participant was provided with a light lunch and was then free to leave the laboratory.

### Measurements

#### Plasma glucose and hormone analyses

Blood samples were collected into ice-chilled tubes containing ethylenediaminetetraacetic acid. Plasma was obtained by centrifuging samples at ~1832*g* force for 15 minutes at 4 °C within 15 minutes of collection and stored at −80 °C until subsequent analysis. Because “total” hormone concentrations were measured, the addition of a protease inhibitor was not required.

Plasma ghrelin concentrations (pg/mL) were measured by an in-house radioimmunoassay using an antibody purchased from Peninsula Laboratories (Cat# T-4745, RRID: AB_518360). The minimum detectable concentration was 40 pg/mL, intra- and interassay coefficients of variation (CVs) were 8.2% and 10.6%, respectively.

Plasma CCK concentrations (pmol/L) were measured by an in-house radioimmunoassay, developed by Prof Rehfeld, using an antiserum (Cat# 92128, RRID: AB_2893008), which binds the circulating bioactive forms of CCK with equal potency, and without cross-reactivity with homologous gastrin peptides (27). The minimum detectable concentration was 0.1 pmol/L, and intra- and interassay CVs were 5% and 15%, respectively.

Plasma PYY concentrations (pmol/L) were measured by an in-house radioimmunoassay using an antiserum against human PYY (1-36), kindly donated by Dr Bärbel Otto, Ludwig-Maximilians-Universität, Munich, Germany (Cat# PYY, RRID: AB_2895649) (28). The minimum detectable concentration was 1.5 pmol/L, and intra- and interassay CVs were 7.9% and 12.9%, respectively.

Plasma total GLP-1 concentrations (pmol/L) were measured by a commercial radioimmunoassay (Millipore Cat# GLPIT-36HK, RRID: AB_2757816). The minimum detectable concentration was 3 pmol/L, and intra- and interassay CVs were 6.9% and 10.9%, respectively.

Plasma insulin concentrations (mU/L) were measured by a commercial ELISA immunoassay (Mercodia Cat# 10-1113, RRID: AB_2877672). The sensitivity of the assay was 1.0 mU/L, and intra- and interassay CVs were 2.9% and 11.6%, respectively.

Plasma glucagon concentrations (pg/mL) were measured by a commercial radioimmunoassay (Millipore Cat# GL-32K, RRID: AB_2757819). The minimum detectable concentration was 15 pg/mL, and intra- and interassay CVs were 3.8% and 9.3%, respectively.

Plasma glucose concentrations (mmol/L) were measured by the glucose oxidase method, using a glucose analyzer (YSI 2300 Stat Plus, Yellow Springs Instruments, Yellow Springs, OH).

#### Antropyloroduodenal pressures

Antropyloroduodenal pressures were digitized and recorded using a computer-based system running commercially available software (MMS Data base software, version 8.17; Solar GI). Data were analyzed for the number and amplitude of antral and duodenal pressures, and isolated pyloric pressure waves (IPPWs) using custom-written software modified to our requirements (A Smout, University Medical Centre, Amsterdam, Netherlands) as described (29). Antral pressure waves and IPPWs were defined by an amplitude of ≥ 10 mmHg with a minimum interval of 10 seconds between peaks. Duodenal pressure waves were defined by an amplitude of ≥ 10 mmHg with a minimum of 3 seconds between peaks (30).

#### GI symptoms

Bloating and nausea were evaluated using a VAS questionnaire (21). The strength of each perception was rated on a 100-mm horizontal line, where 0 mm represented “sensation not felt at all” and 100 mm “sensation felt the greatest”. Subjects were asked to indicate how they were feeling at each time point by placing a vertical mark on the line.

### Data and Statistical Analysis

Statistical analysis was performed using SPSS software (version 27.0; IBM, Chicago, IL, USA).

Plasma CCK, PYY, GLP-1, insulin, glucagon, and VAS data were summarized by areas under the curve (AUC), and ghrelin and glucose data, whose concentrations, in contrast to the above hormones, were anticipated to be reduced by administration of quinine, using inverted incremental AUC (iAUCinv), calculated using the trapezoidal rule. Numbers and amplitudes of antral and duodenal pressure waves were used to calculate antral and duodenal motility indexes (MIs), using the following equation: MI (mmHg) = natural logarithm {[sum of amplitudes × number of pressure waves] + 1} (31). Numbers and amplitudes of IPPWs were expressed as total numbers and mean amplitudes, respectively. All data were summarized for both the entire study period, namely, AUC_0-120_, MI_0-120_, and number/amplitude_0-120_, as well as for the first 60 minutes, namely, AUC_0-60_, etc, and for the second 60 minutes, namely, AUC_60-120_, etc, to evaluate whether effects were evident predominantly “earlier” or “later”, based on our previous findings (32-34). We also determined peak values, as well as times to peak values, for plasma CCK and insulin concentrations, and the number of IPPWs. These were not calculated for other parameters, given that it was shown subsequently that these did not reach a peak (or nadir, in the case of ghrelin), but continued to rise (or fall), throughout the study period.

One-way analysis of variance (ANOVA) was used to evaluate effects of treatments over time relative to baseline. In order to evaluate the potential effects of both site of administration and sex, data were analyzed using mixed effects maximum likelihood models with visit number (1 or 2), site of administration, sex, and the site-by-sex interaction as fixed factors, and a random subject effect. An unstructured covariance matrix was used to account for the repeated visits per subject. Prespecified contrasts of the model-estimated marginal means were calculated for the 4 primary comparisons of interest: male ID vs male IG, female ID vs female IG, female ID vs male ID, female IG vs male IG. No adjustments for multiple comparisons were made, because the 4 contrasts were prespecified (ie, before examination of the data) to correspond to the primary research hypotheses. They were interpreted independently, regardless of the significance of the other contrasts or the significance of the overall effects from the mixed models. All data are reported as mean ± standard error of the mean (SEM).

## Results

The QHCl treatments were well tolerated, and all participants completed the study visits without reporting any adverse effect. Oral detection thresholds for QHCl were 0.10 ± 0.07 mmol/L in males, and 0.10 ± 0.02 mmol/L in females, with no difference between the 2 groups. No participant experienced hypoglycemia in response to QHCl, and glucose concentrations remained above 3.9 mmol/L in all participants. There were no differences in fasting plasma concentrations of gut hormones (Table 1), pancreatic hormones and glucose (Table 2), or antropyloroduodenal pressures (Table 3).

**Table 1.** Plasma ghrelin, CCK, PYY, and GLP-1 concentrations at baseline and following intragastric or intraduodenal administration of quinine in healthy men and women

	Male			Female		
	IG-QHCl	ID-QHCl	*P* values	IG-QHCl	ID-QHCl	*P* values
**Plasma ghrelin**						
Fasting concentration, pg/mL	1499 ± 160	1560 ± 163	0.243	1643 ± 214	1645 ± 200	0.989
iAUCinv_0-120min_, pg/mL × min	5724 ± 5693	19894 ± 5276	0.006	15133 ± 5442	28384 ± 5412	0.007
iAUCinv_0-60min_, pg/mL × min	2940 ± 1891	7550 ± 1739	0.019	4983 ± 1795	9673 ± 1795	0.013
AUCinv_60-120min_, pg/mL × min	2468 ± 4189	12197 ± 3873	0.010	10292 ± 4002	19032 ± 4002	0.015
**Plasma CCK**						
Fasting concentration, mmol/L	1.0 ± 0.1	1.0 ± 0.2	0.407	0.7 ± 0.2	0.7 ± 0.2	0.430
AUC_0-120min_, pmol/L × min	174 ± 20	261 ± 20	0.002	199 ± 20	234 ± 20	0.147
AUC_0-60min_, pmol/L × min	85 ± 13	148 ± 13	0.006	105 ± 13	151 ± 13	0.030
AUC_60-120min_, pmol/L × min	91 ± 9	107 ± 9	0.134	92 ± 9	85 ± 9	0.538
Peak concentration, pmol/L	2.4 ± 0.4	3.8 ± 0.4	0.010	3.1 ± 0.4	4.2 ± 0.4	0.050
Time to peak concentration, min	33 ± 5	18 ± 5	0.020	42 ± 5	25 ± 5	0.010
**Plasma PYY**						
Fasting concentration, pmol/L	32.2 ± 3.6	33.9 ± 2.6	0.199	29.9 ± 3.9	29.4 ± 4.3	0.920
AUC_0-120min_, pmol/L × min	4215 ± 239	5068 ± 232	0.017	3983 ± 235	4879 ± 239	0.013
AUC_0-60min_, pmol/L × min	1973 ± 102	2390 ± 99	0.010	1902 ± 100	2268 ± 101	0.022
AUC_60-120min_, pmol/L × min	2242 ± 154	2669 ± 149	0.048	2090 ± 151	2609 ± 154	0.020
**Plasma GLP-1**						
Fasting concentration, pmol/L	11.5 ± 1.3	12.0 ± 1.5	0.283	9.8 ± 1.10	9.7 ± 1.0	0.458
AUC_0-120min_, pmol/L × min	1480 ± 99	1808 ± 99	0.025	1377 ± 98	1685 ± 99	0.034
AUC_0-60min_, pmol/L × min	675 ± 43	819 ± 43	0.028	623 ± 43	746 ± 43	0.054
AUC_60-120min_, pmol/L × min	791 ± 66	985 ± 66	0.040	739 ± 66	926 ± 66	0.046

Data are means ± SEMs. n = 14 in each group, except for ghrelin (n = 12).

Abbreviations: AUC, area under the curve; CCK, cholecystokinin; GLP-1, glucagon-like peptide-1; iAUCinv, inverted incremental AUC; ID-QHCl, intraduodenal quinine hydrochloride; IG-QHCl, intragastric quinine hydrochloride; PYY, peptide-YY. There were no effects of sex on plasma ghrelin, CCK, PYY, or GLP-1 after either IG-QHCl or ID-QHCl.

**Table 2.** Plasma insulin, glucagon, and glucose concentrations at baseline and following intragastric or intraduodenal administration of quinine in healthy, normal-weight men and women

	Male			Female		
	IG-QHCl	ID-QHCl	*P* values	IG-QHCl	ID-QHCl	*P* values
**Plasma insulin**						
Fasting concentration, mU/L	2.7 ± 0.5	2.4 ± 0.4	0.288	3.6 ± 0.5	3.3 ± 0.2	0.463
AUC_0-120min_, mU/L × min	648 ± 77	865 ± 79	0.024	754 ± 79	1042 ± 77	0.004
AUC_0-60min_, mU/L × min	261 ± 52	485 ± 53	0.006	344 ± 53	663 ± 52^a^	0.001
AUC_60-120min_, mU/L × min	370 ± 41	368 ± 42	0.956	423 ± 42	394 ± 41	0.462
Peak concentration, mU/L	8.5 ± 1.5	13.2 ± 1.5	0.040	11.7 ± 1.5	19 ± 1.5^b^	0.002
Time to peak concentration, min	71 ± 5	39 ± 5	0.001	60 ± 5	39 ± 5	0.012
**Plasma glucagon**						
Fasting concentration, pg/mL	41 ± 4	43 ± 4	0.941	40 ± 2	41 ± 4	0.777
AUC_0-120min_, pg/mL × min	5044 ± 371	6189 ± 362	0.030	5401 ± 362	6607 ± 362	0.021
AUC_0-60min_, pg/mL × min	2193 ± 138	2710 ± 139	0.023	2427 ± 137	2800 ± 137	0.081
AUC_60-120min_, pg/mL × min	2842 ± 250	3497 ± 244	0.043	2990 ± 245	3780 ± 245	0.015
**Plasma glucose**						
Fasting concentration, mmol/L	4.8 ± 0.1	4.5 ± 0.1	0.251	5.3 ± 0.1	5.4 ± 0.1	0.487
iAUCinv_0-120min_, mmol/L × min	57 ± 9	58 ± 8	0.947	50 ± 8	99 ± 8^c^	0.001
iAUCinv_0-60min_, mmol/L × min	21 ± 4	25 ± 4	0.505	9 ± 4^d^	39 ± 4^e^	0.001
iAUCinv_60-120min_, mmol/L × min	36 ± 6	33 ± 6	0.634	41 ± 6	59 ± 6^f^	0.036

Data are means ± SEMs. n = 14 in each group, except for glucagon (n = 12).

Abbreviations: AUC, area under the curve; iAUCinv, inverted incremental AUC; ID-QHCl, intraduodenal quinine hydrochloride; IG-QHCl, intragastric quinine hydrochloride.

Differences from respective values in males:

^a^ *P* = 0.021,

^b^ *P* = 0.010,

^c^ *P* = 0.001,

^d^ *P* = 0.040,

^e^ *P* = 0.011,

^f^ *P* = 0.003.

**Table 3.** Motility index of antral and duodenal pressure waves, and number, peak, and the time to peak number of isolated pyloric pressure following intragastric or intraduodenal administration of quinine in healthy men and women

	Male			Female		
	IG-QHCl	ID-QHCl	*P* values	IG-QHCl	ID-QHCl	*P* values
**Antral pressure waves**						
MI_0-120min_, mmHg	11 ± 1	10 ± 1	0.625	11 ± 1	11 ± 1	0.999
MI_0-60min_, mmHg	10 ± 1	8 ± 1	0.014	11 ± 1	11 ± 1^a^	0.703
MI_60-120min_, mmHg	7 ± 1	7 ± 1	0.687	10 ± 1	9 ± 1^b^	0.420
**Isolated pyloric pressure waves**						
Total number_0-120min_	25 ± 10	36 ± 11	0.426	16 ± 6	56 ± 15	0.002
Total number_0-60min_	11 ± 4	22 ± 8	0.050	6 ± 2	30 ± 9	0.013
Total number_60-120min_	14 ± 6	14 ± 4	0.451	9 ± 4	26 ± 9	0.039
Peak number	7 ± 2	10 ± 2	0.356	6 ± 2	16 ± 3	0.001
Time to peak number, min	70 ± 9	47 ± 10	0.173	90 ± 5	50 ± 8	0.008
Mean amplitude_0-120min_, mmHg	18 ± 4	18 ± 3	0.345	21 ± 3	20 ± 2	0.333
Mean amplitude_0-60min_, mmHg	21 ± 3	21 ± 4	0.533	20 ± 3	22 ± 3	0.451
Mean amplitude_60-120min_, mmHg	21 ± 5	20 ± 3	0.613	24 ± 3	23 ± 3	0.517
**Duodenal pressure waves**						
MI_0-120min_, mmHg	15 ± 0	16 ± 0	0.404	15 ± 0	16 ± 0	0.032
MI_0-60min_, mmHg	13 ± 0	14 ± 0	0.042	14 ± 0	15 ± 0^c^	0.007
MI_60-120min_, mmHg	14 ± 0	14 ± 0	0.835	15 ± 0	15 ± 0	0.242

Data are means ± SEMs. n = 14 in each group.

Abbreviations: ID-QHCl, intraduodenal quinine hydrochloride; IG-QHCl, intragastric quinine hydrochloride; MI, motility index (calculated as MI (mmHg) = natural logarithm {[sum of amplitudes × number of pressure waves] + 1}).

Differences from respective values in males:

^a^ *P* = 0.011,

^b^ *P* = 0.045,

^c^P = 0.017.

### Gut Hormones

#### Plasma ghrelin concentrations

**Figure 1.**
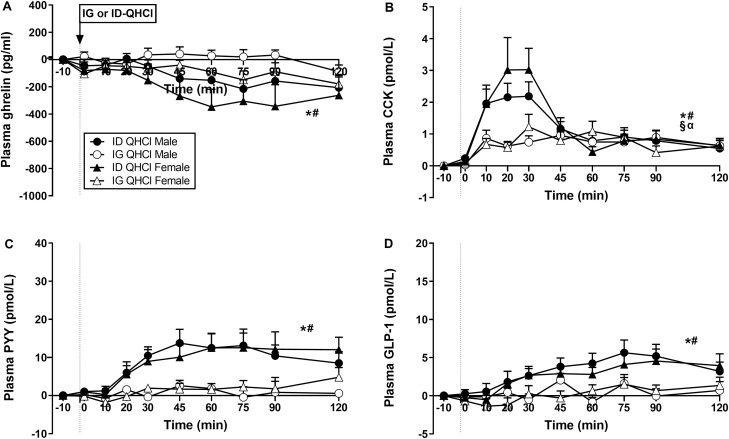
Plasma concentrations of (A) ghrelin, (B) cholecystokinin (CCK), (C) peptide-YY (PYY) and (D) glucagon-like peptide-1 (GLP-1) following quinine hydrochloride (QHCl), administered (at t = −1 min) either intraduodenally (ID) or intragastrically (IG), in a dose of 600 mg, in 14 healthy men and 14 healthy women. One-way ANOVA was used to evaluate effects of treatments over time relative to baseline. (A) Time effects in * males (*P* = 0.034) and # females (*P* = 0.035) after ID-QHCl, (B) time effects in * males (*P* = 0.001) and # females (*P* = 0.004) after ID-QHCl, time effects in § males (*P* = 0.001) and α females (*P* = 0.024) after IG-QHCl, (C) time effects in * males (*P* = 0.001) and # females (*P* = 0.005) after ID-QHCl, (D) time effects in * males (*P* = 0.001) and # females (*P* = 0.010) after ID-QHCl. Data are means ± SEM at each sampling time point and expressed as changes from baseline.

While there were effects of time on plasma ghrelin after ID-QHCl, but not IG-QHCl, in both males (*P* = 0.034) and females (*P* = 0.035), and mean levels after ID-QHCl appeared to be lower compared with baseline, post hoc analysis revealed no differences (Fig. 1A).

There were effects of site on plasma ghrelin (Table 1, Fig. 1A). Ghrelin iAUCinv_0-120,_ iAUCinv_0-60_, and iAUCinv_60-120_ were greater after ID-QHCl compared with IG-QHCl in both males (iAUCinv_0-120_: *P* = 0.006, iAUCinv_0-60_: *P* = 0.019, iAUCinv_60-120_: *P* = 0.010) and females (iAUCinv_0-120_: *P* = 0.007, iAUCinv_0-60_: *P* = 0.013, iAUCinv_60-120_: *P* = 0.015).

#### Plasma CCK concentrations

There were effects of time on plasma CCK after ID-QHCl and IG-QHCl, with peak concentrations occurring within 20 to 30 minutes (Fig. 1B). Plasma CCK was greater after ID-QHCl compared with baseline, from t = 10 to 120 minutes in males (time effect: *P* = 0.001), and from t = 10 to 45 minutes and at t = 75 minutes in females (time effect: *P* = 0.004). Moreover, plasma CCK was greater after IG-QHCl, compared with baseline, from t = 10 to 45 minutes and t = 75 to 90 minutes in males (time effect: *P* = 0.001), and from t = 10 to 30 minutes and at t = 60 and 120 minutes in females (time effect: *P* = 0.024).

There were effects of site on plasma CCK (Table 1, Fig. 1B). CCK AUC_0-120_ and AUC_0-60_ were greater after ID-QHCl compared with IG-QHCl in males (AUC_0-120_: *P* = 0.002, AUC_0-60_: *P* = 0.006). Moreover, AUC_0-60,_ but not AUC_0-120_ or AUC_60-120_, was greater after ID-QHCl compared with IG-QHCl in females (AUC_0-60_: *P* = 0.030). There were also effects of site on the peak CCK concentration, and the time to peak CCK concentration. Peak concentrations were greater (males: *P* = 0.01; females: *P* = 0.05), and times to peak concentration less (males: *P* = 0.02; females: *P* = 0.010), after ID-QHCl compared with IG-QHCl, in both males and females.

#### Plasma PYY concentrations

There were effects of time on plasma PYY after ID-QHCl, but not IG-QHCl (Fig. 1C). Plasma PYY was greater after ID-QHCl, compared with baseline, between t = 30 and 120 minutes in males (time effect: *P* = 0.001), and at t = 60 and 120 minutes in females (time effect: *P* = 0.005).

There were effects of site on plasma PYY (Table 1, Fig. 1C). PYY AUC_0-120,_ AUC_0-60_ and AUC_60-120_ were greater after ID-QHCl compared with IG-QHCl in both males (AUC_0-120_: *P* = 0.017, AUC_0-60_: *P* = 0.010; AUC_60-120_: *P* = 0.048) and females (AUC_0-120_: *P* = 0.013, AUC_0-60_: *P* = 0.022; AUC_60-120_: *P* = 0.020).

#### Plasma GLP-1 concentrations

There were effects of time on plasma GLP-1 after ID-QHCl, but not IG-QHCl (Fig. 1D). Plasma GLP-1 was greater after ID-QHCl, compared with baseline, between t = 45 and 120 minutes in males (time effect: *P* = 0.001) and in females (time effect: *P* = 0.010).

There were effects of site on plasma GLP-1 (Table 1, Fig. 1D). GLP-1 AUC_0-120,_ AUC_0-60_, and AUC_60-120_ were greater after ID-QHCl compared with IG-QHCl in both males (AUC_0-120_: *P* = 0.025, AUC_0-60:_*P* = 0.028, AUC_60-120_: *P* = 0.040) and females (AUC_0-120_: *P* = 0.034, AUC_0-60_: *P* = 0.054, AUC_60-120_: *P* = 0.046).

There were no effects of sex on plasma ghrelin, CCK, PYY, or GLP-1 after either IG-QHCl or ID-QHCl (Table 1, Figs. 1A-1D).

### Pancreatic Hormones and Plasma Glucose

#### Plasma insulin concentrations

**Figure 2.**
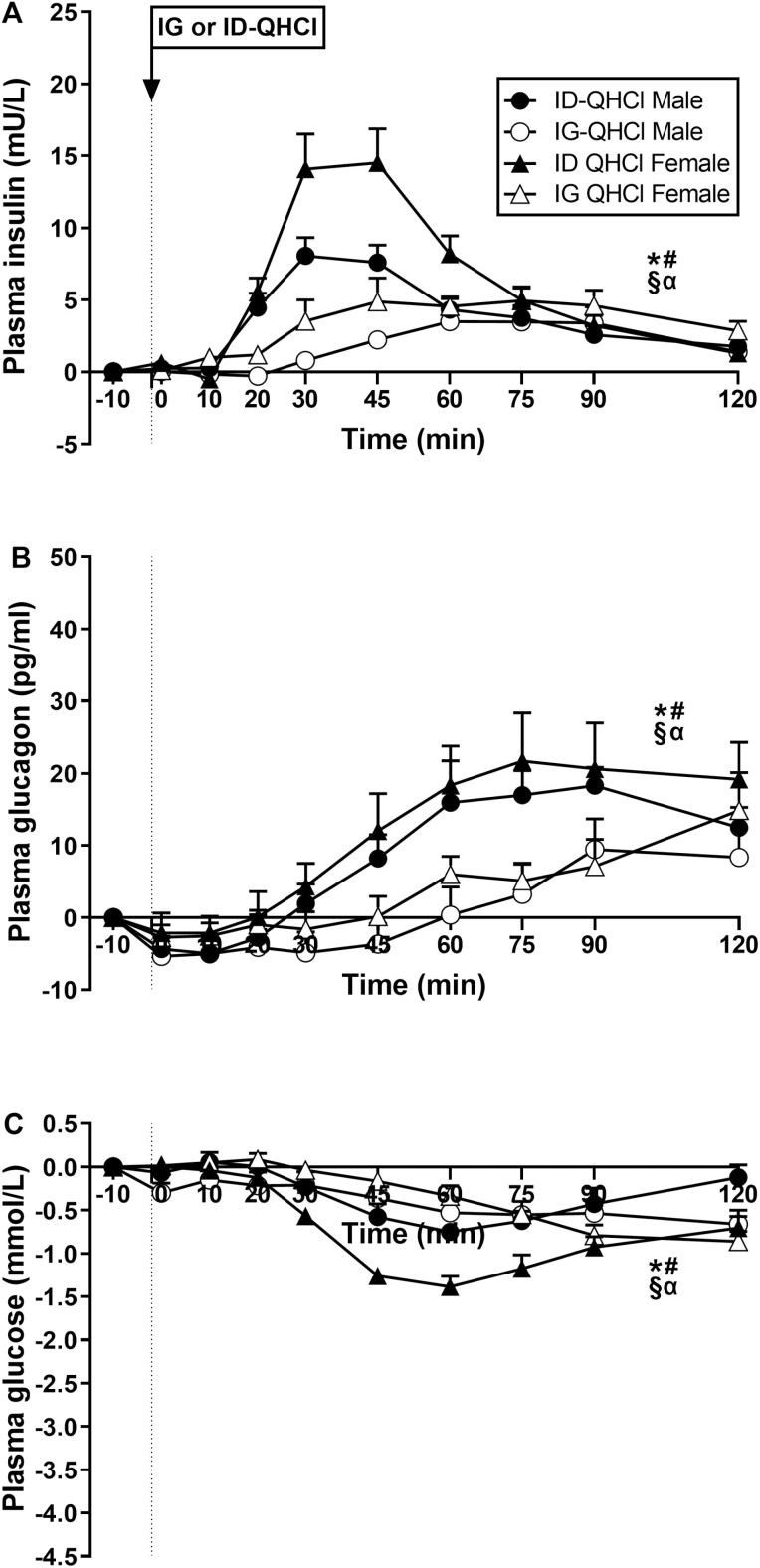
Plasma concentrations of (A) insulin, (B) glucagon and (C) glucose following quinine hydrochloride (QHCl), administered (at t = −1 min) either intraduodenally (ID) or intragastrically (IG), in a dose of 600 mg, in 14 healthy men and 14 healthy women. One-way ANOVA was used to evaluate effects of treatments over time relative to baseline. (A) Time effects in * males (*P* = 0.001) and # females (*P* = 0.001) after ID-QHCl, time effects in § males (*P* = 0.001) and α females (*P* = 0.010) after IG-QHCl, (B) time effects in * males (*P* = 0.001) and # females (*P* = 0.001) after ID-QHCl, time effects in § males (*P* = 0.001) and α females (*P* = 0.002) after IG-QHCl, (C) time effects in * males (*P* = 0.001) and # females (*P* = 0.001) after ID-QHCl, time effects in § males (*P* = 0.016) and α females (*P* = 0.001) after IG-QHCl. Data are means ± SEM at each sampling time point and expressed as changes from baseline.

There were effects of time on plasma insulin after ID-QHCl and IG-QHCl (Fig. 2A). Plasma insulin was greater after ID-QHCl compared with baseline, between t = 20 and 120 minutes in males (time effect: *P* = 0.001) and females (time effect: *P* = 0.001). Moreover, plasma insulin was greater after IG-QHCl, compared with baseline, between t = 45 and 75 minutes in males (time effect: *P* = 0.001), and between t = 60 and 120 minutes in females (time effect: *P* = 0.010).

There were effects of site on plasma insulin (Table 2, Fig. 2A). Insulin AUC_0-120_ and AUC_0-60_, but not AUC_60-120_, were greater after ID-QHCl compared with IG-QHCl in both males (AUC_0-120_: *P* = 0.024, AUC_0-60_: *P* = 0.006) and females (AUC_0-120_: *P* = 0.004, AUC_0-60_: *P* = 0.001). There were also effects of site on peak insulin, and the time to peak insulin concentration. Peak concentrations were greater (males: *P* = 0.040; females: *P* = 0.002), and time to peak concentration less (males: *P* = 0.001; females: *P* = 0.012), after ID-QHCl compared with IG-QHCl in both males and females.

There were effects of sex on plasma insulin (Table 2, Fig. 2A). Insulin AUC_0-60_, but not AUC_0-120_ or AUC_60-120_, was greater in females than in males after ID-QHCl (AUC_0-60_: *P* = 0.021), but not IG-QHCl. There was also an effect of sex on peak insulin, but not time to peak insulin concentration, after ID-QHCl, but not IG-QHCl. Peak concentration after ID-QHCl was greater in females compared with males (*P* = 0.01).

#### Plasma glucagon concentrations

There were effects of time on plasma glucagon after ID-QHCl and IG-QHCl (Fig. 2B). Plasma glucagon was greater after ID-QHCl between t = 60 and 120 minutes in males (time effect: *P* = 0.001) and females (time effect: *P* = 0.001), and after IG-QHCl in males (time effect: *P* = 0.001) and females (time effect: *P* = 0.002).

There were effects of site on plasma glucagon (Table 2, Fig. 2B). Glucagon AUC_0-120_, AUC_0-60_, and AUC_60-120_ were greater after ID-QHCl compared with IG-QHCl in both males (AUC_0-120_: *P* = 0.030, AUC_0-60_: *P* = 0.023, AUC_60-120_: *P* = 0.043) and females (AUC_0-120_: *P* = 0.020, AUC_0-60_: *P* = 0.081, AUC_60-120_: *P* = 0.015).

There were no effects of sex on plasma glucagon after IG-QHCl or ID-QHCl (Table 2, Fig. 2B).

#### Plasma glucose concentrations

There were effects of time on plasma glucose after ID-QHCl and IG-QHCl (Fig. 2C). Plasma glucose was less after ID-QHCl, compared with baseline, between t = 45 and 75 minutes in males (time effect: *P* = 0.001) and between t = 30 and 120 minutes in females (time effect: *P* = 0.001). Moreover, plasma glucose was less after IG-QHCl, compared with baseline, between t = 60 and 120 minutes in males (time effect: *P* = 0.016) and between t = 75 and 120 minutes in females (time effect: *P* = 0.001).

There were effects of site on plasma glucose (Table 2, Fig. 2C). Plasma glucose iAUCinv_0-120_, iAUCinv_0-60_, and iAUCinv_60-120_, were greater after ID-QHCl compared with IG-QHCl in females (iAUCinv_0-120_: *P* = 0.001, iAUCinv_0-60_: *P* = 0.001, iAUCinv_60-120_: *P* = 0.036). In contrast, there were no differences after ID-QHCl compared with IG-QHCl in males.

There were effects of sex on plasma glucose (Table 2, Fig. 2C). Plasma glucose iAUCinv_0-120_, iAUCinv_0-60_, and iAUCinv_60-120_ were greater in females than in males after ID-QHCl (iAUCinv_0-120_: *P* = 0.001, iAUCinv_0-60_: *P* = 0.011, iAUCinv_60-120_: *P* = 0.003), while iAUCinv_0-60_, but not iAUCinv_0-120_ or iAUCinv_60-120_, was less in females than in males after IG-QHCl (iAUCinv_0-60_: *P* = 0.04).

### Antropyloroduodenal Pressures

#### Antral pressures

There was an effect, albeit modest, of site on antral MI. Antral MI_0-60_, but not MI_0-120_ or MI_60-120_, was greater after IG-QHCl compared with ID-QHCl in males (*P* = 0.014), but not in females (Table 3).

There were effects, albeit modest, of sex on antral MI. Antral MI_0-60_ (*P* = 0.011) and MI_60-120_ (*P* = 0.045), but not MI_0-120_, were greater in females than in males after ID-QHCl but not IG-QHCl (Table 3).

#### Isolated pyloric pressure waves

**Figure 3.**
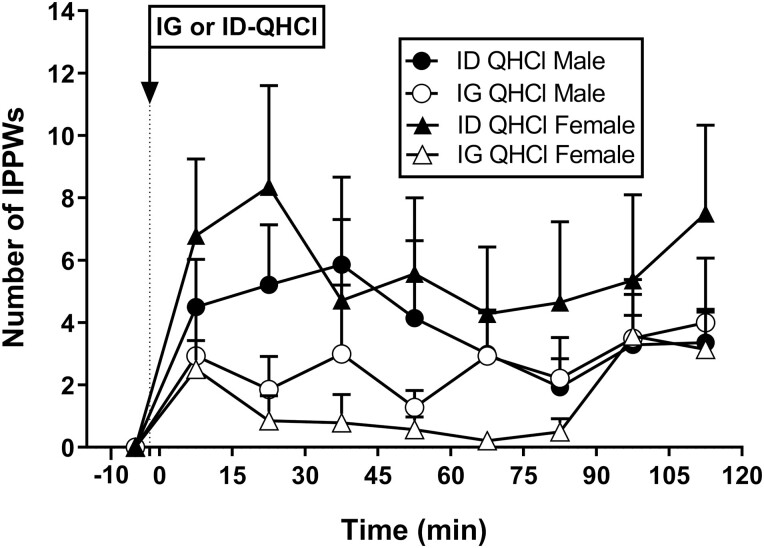
Number of isolated pyloric pressure waves following quinine hydrochloride (QHCl), administered (at t = −1 min) either intraduodenally (ID) or intragastrically (IG), in a dose of 600 mg, in 14 healthy men and 14 healthy women. One-way ANOVA was used to evaluate effects of treatments over time relative to baseline. Data are means ± SEM for baseline (t = -1 to 0 min) and 15-min intervals following QHCl administration and expressed as changes from baseline.

There were no effects of time on the number, or amplitude, of IPPWs after IG-QHCl or ID-QHCl in males or females (Fig. 3). The IPPW response to ID-QHCl peaked between 15 and 45 minutes.

There was an effect of site on the number, but not the amplitude, of IPPWs (Table 3, Fig. 3). In females, IPPW number_0-120_, number_0-60_, and number_60-120_ were greater after ID-QHCl compared with IG-QHCl (number_0-120_: *P* = 0.002, number_0-60_: *P* = 0.013, number_60-120_: *P* = 0.039). In males, IPPW number_0-60_, but not number_0-120_ or number_60-120_, was greater after ID-QHCl compared with IG-QHCl (number_0-60_: *P* = 0.050). There were also effects of site on the peak number and the time to peak number of IPPWs. Peak number was greater (*P* = 0.001), and time to peak number less (*P* = 0.008), after ID-QHCl than IG-QHCl in females, but not males.

There were no effects of sex on the number or amplitude of IPPWs after IG-QHCl or ID-QHCl, although mean values appeared to be greater in females than males (Table 3, Fig. 3).

#### Duodenal pressures

There were effects, albeit modest, of site on duodenal MI. In females, duodenal MI_0-120_ and MI_0-60_, but not MI_60-120_, were greater after ID-QHCl compared with IG-QHCl (MI_0-120_: *P* = 0.032, MI_0-60_: *P* = 0.007). In males, the duodenal MI_0-60_, but not MI_0-120_ and MI_60-120_, was greater after ID-QHCl compared with IG-QHCl (*P* = 0.042) (Table 3).

There were effects, albeit modest, of sex on duodenal MI. The duodenal MI_0-60_ was greater in females than in males after ID-QHCl (*P* = 0.017), and after IG-QHCl (*P* = 0.075). The duodenal MI_60-120_ was also greater in females than in males after ID-QHCl (*P* = 0.061), but not IG-QHCl (Table 3).

### GI Symptoms

#### Bloating

**Figure 4.**
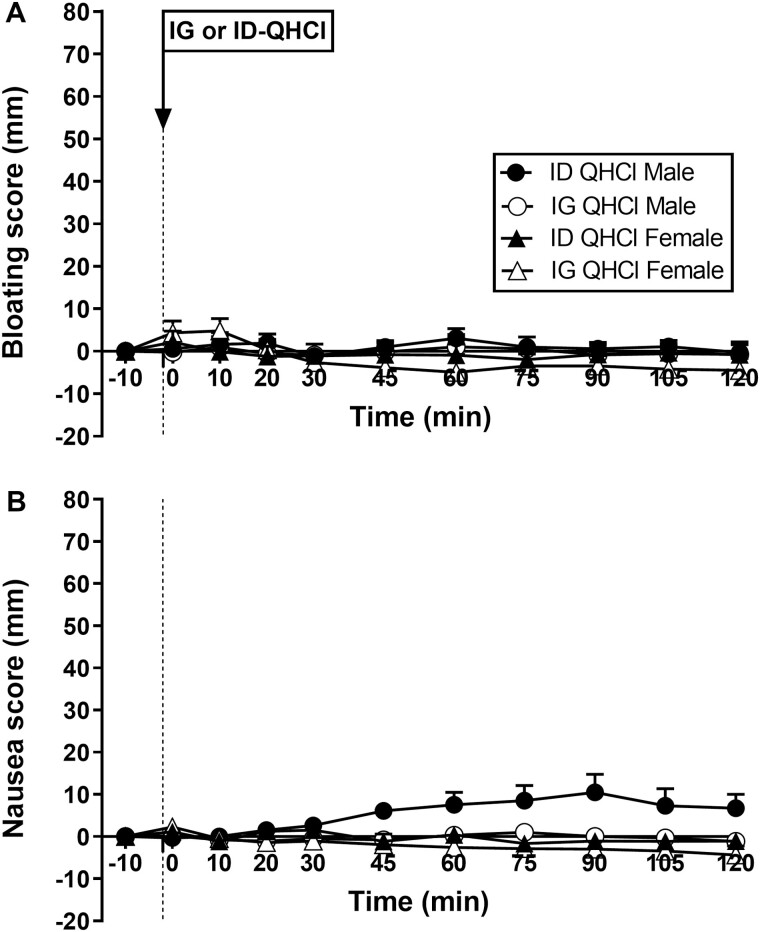
Scores for bloating (A) and nausea (B) following quinine hydrochloride (QHCl), administered (at t = -1 min) either intraduodenally (ID) or intragastrically (IG), in a dose of 600 mg, in 14 healthy men and 14 healthy women. One-way ANOVA was used to evaluate effects of treatments over time relative to baseline. Data are means ± SEM at each time point and expressed as changes from baseline.

There were no effects of time, site, or sex on ratings of bloating after either IG-QHCl or ID-QHCl in males or females (Fig. 4A).

#### Nausea

There were no effects of time on nausea after either IG-QHCl or ID-QHCl in males or females (Fig. 4B).

There was an effect of site on nausea (Fig. 4B). In males, mean nausea was greater after ID-QHCl compared with IG-QHCl (*P* = 0.004). The slight increase in nausea was due to the ratings in 2 participants, which increased by ~40 mm, while no other participants reported any nausea.

There was an effect of sex on nausea (Fig. 4B), which was greater in males than in females after ID-QHCl (*P* = 0.010), but not IG-QHCl.

## Discussion

Our study provides a comprehensive evaluation of the comparative effects of IG and ID administration of QHCl, in a dose of 600 mg, on GI functions that are integral to the regulation of energy intake and blood glucose in healthy men and women, including gut and pancreatic hormones and pyloric pressures; the latter underlie the regulation of gastric emptying. Key findings are that (1) the effects of QHCl on gut and pancreatic hormones, glucose, and pyloric pressures were affected markedly by the site of its administration, with ID quinine being much more potent, and (2) the majority of these effects, with the exception of those relating to insulin and glucose, were not influenced by sex. These insights have important implications for an understanding of the factors modulating the effects of quinine, and probably other bitter substances, on GI functions.

That ID-QHCl had greater effects to modulate gut and pancreatic hormones, reduce glucose and stimulate pyloric pressures, suggests that the regulation of these effects originates primarily in the small intestine. We have shown previously that ID- and IG-QHCl, when administered 30 minutes and 60 minutes, respectively, before a mixed-nutrient drink, have comparable effects to stimulate plasma GLP-1 (16), suggesting that, in both situations, sufficient interaction of QHCl with small intestinal bitter receptors was achieved. Our current findings support this hypothesis and are also consistent with animal studies showing a greater density of bitter taste receptors in the duodenum than the stomach (6, 18). Because quinine appears to be absorbed primarily in the small intestine (35, 36), IG-QHCl also reaches the small intestine, although with a delay, and due to dilution, almost certainly in lower concentration than ID-QHCl. Moreover, a shorter length of intestine may be exposed following IG-QHCl. Both concentration and length of intestine exposed are known determinants of the effects of dietary nutrients on gut hormone release, gastric emptying, and energy intake (37-39), and may underlie the more potent effects of ID vs IG-QHCl.

Bitter taste receptors are present throughout the GI tract on enteroendocrine cells (1, 3, 6, 39).

CCK is produced primarily in the proximal small intestine, in contrast to GLP-1 and PYY, which are released predominantly more distally (40, 41). Hence, the patterns of plasma concentrations of these hormones in response to quinine in the GI lumen would also be expected to vary. Accordingly, the rise in plasma CCK occurred within ~10 minutes and peaked within ~20-30 minutes. In contrast, the rises in GLP-1 and PYY were gradual and sustained for at least 120 minutes. These patterns of release are consistent with the effects of nutrients, such as fatty acids and protein (34, 42). Of note, CCK, but not GLP-1 or PYY, was stimulated by IG-QHCl, albeit less than ID-QHCl, underscoring the importance of the length of exposure and that IG-QHCl may not readily reach the distal small intestine, the major site of release for GLP-1 and PYY. While ghrelin is primarily secreted from the stomach (41), the observed greater suppression of plasma ghrelin after ID-QHCl, compared with IG-QHCl (which was ineffective), is indicative of an important contribution emanating from the small intestine. This is supported by the finding that small intestinal, but not gastric, exposure to nutrient (glucose) is sufficient to suppress plasma ghrelin (43). The recent report of a lack of effect of ID- or IG-QHCl on either plasma CCK or ghrelin may reflect the lower dose used (17), indicating dose-dependency. The suppressive effect of small intestinal nutrients or quinine on ghrelin release may, at least in part, be mediated by hormones. Intravenous infusions of CCK or PYY (3-36) reduce plasma ghrelin levels in humans (44-46); moreover, involvement of PYY may potentially underlie the continued suppression after 60 minutes.

Both ID and IG-QHCl stimulated insulin; however, ID-QHCl was much more potent. While the mechanisms underlying quinine-induced stimulation of insulin remain to be elucidated, GLP-1 may potentially have played a role, which could be clarified by studies using a specific GLP-1 receptor antagonist. GLP-1-induced insulin secretion is known to be glucose-dependent—below a plasma glucose of 4.3 mmol/L, the insulinotropic effect of intravenous GLP-1, even at supraphysiological concentrations, is negligible (47). Alternatively, quinine may stimulate insulin following absorption into the bloodstream by activating receptors on pancreatic beta cells directly. Preclinical studies have demonstrated quinine-induced insulin secretion from isolated pancreatic beta cells (48). In our study, the rise in insulin was associated with subsequent reduction in plasma glucose by up to ~1.5 mmol/L, as well as stimulation of glucagon. Whether the observed rise in glucagon was a direct effect of quinine or triggered in response to glucose lowering, is uncertain, although the observed time course is more consistent with the concept that glucagon release followed the stimulation of insulin.

The effect of quinine on pyloric pressures was also site-dependent, with ID more potent than IG administration. Given the key role of pyloric pressures in the regulation of gastric emptying (49), this finding is consistent with our previous observations that IG quinine only slowed gastric emptying when it was given 60 minutes (16), but not 30 minutes (13), before a drink. Thus, these data provide compelling evidence for a key role of the small intestine in mediating the effects of quinine on pyloric motility. The pattern of pyloric pressures, with peak stimulation occurring during the first 15 to 45 minutes, reflected the pattern of CCK release. Since CCK receptors are located on pyloric smooth muscle cells (50), the effect of quinine on pyloric pressures was likely mediated, at least in part, by endogenous CCK. Site-dependency of effects on antral and duodenal pressures was also evident, with a greater effect of IG quinine on antral, and a greater effect of ID quinine on duodenal, pressures, consistent with direct local effects of quinine on smooth muscle cells. Indeed, some bitter substances (e.g., denatonium benzoate, chloroquine) enhance motility by stimulating bitter taste receptors located on muscle strips of mouse antrum directly (1). However, the magnitude of effects on antral and duodenal pressures in our study was small.

The GI effects of bitter substances have been suggested to be sex-dependent, based on reports of greater perception of a bitter lingual stimulus (6-n-propylthiouracil) in females than males (19, 51), although we did not find any differences in oral detection thresholds in our study. We have found inverse relationships between the oral perception of fatty acids and the magnitude of stimulation of pyloric pressures, CCK, and PYY in healthy individuals (52). We observed greater effects in females than in males for quinine to stimulate insulin and lower plasma glucose, and modest effects to increase antral and duodenal motility, while no effects of sex on pyloric pressures or ghrelin were evident. Interestingly, a greater insulin response during an oral glucose tolerance test in healthy females, compared with males, has been reported, although in that study, and in contrast to the current findings, glucagon was suppressed more in males (53). Since we found no significant evidence of sex effects on any of the GI hormones or pyloric pressures, our observations do not support the notion of major sex-dependent GI effects of quinine.

Some limitations of our study should be noted. We only evaluated one dose of quinine, which was not adjusted for differences in body weight between men and women. We did not include a control condition and, thus, determined effects of quinine relative to baseline. However, infusion of isotonic saline is known to not affect the parameters we assessed (29, 33, 34, 42). While ID or IG administration cannot be used routinely, the insights may be pivotal for the design of formulations for targeted delivery. We did not measure plasma motilin, which is modulated by bitter substances (14, 15), although IG, but not ID, QHCl appears to suppress motilin (17). We only assessed effects of quinine on fasting glucose; whether quinine lowers postprandial glucose in females more than in males warrants evaluation. Our findings should also be assessed as a function of age, body weight, race/ethnicity, and glucose tolerance status. The study was not powered for analysis of site of administration-by-sex interactions, so, potential synergistic effects between these factors were not assessed. Finally, it is important to recognize that quinine in higher doses may be associated with adverse effects, particularly in the longer-term, and also engage mechanisms other than bitter receptors, including sodium channel blockade. It is, therefore, important to determine whether the observed effects of quinine are also evident at lower doses.

In conclusion, administration of quinine directly into the duodenum has much greater effects to modulate plasma gut and pancreatic hormones and pyloric pressures than intragastric administration, indicating the importance of the interaction of quinine with small intestinal bitter receptors. While our findings do not support the generalized concept of sex-dependent GI effects of quinine, the glucose-lowering effects of quinine appear to be enhanced in females. Taken together, our observations have implications for the use of targeted delivery options and potential for more personalized approaches for the administration of quinine, and possibly broadly, bitter substances, to optimize their potency to stimulate GI functions that modulate energy intake and blood glucose.

## Data Availability

Some or all datasets generated during and/or analyzed during the current study are not publicly available but are available from the corresponding author on reasonable request.

## Abbreviations

ANOVA: analysis of variance
AUC: area under the curve
CCK: cholecystokinin
CV: coefficient of variation
GI: gastrointestinal
GLP-1: glucagon-like peptide-1
ID: intraduodenal
IG: intragastric
IPPW: isolated pyloric pressure wave
MI: motility index
PYY: peptide YY (peptide tyrosine-tyrosine)
QHCl: quinine hydrochloride
VAS: visual analog scale

## Clinical Trial Information

The study was registered as a clinical trial with the Australian and New Zealand Clinical Trials Registry (www.anzctr.org.au; Registration ID: ACTRN12619000707167).

## References

[1] Avau B , Rotondo A, Thijs T, et al Targeting extra-oral bitter taste receptors modulates gastrointestinal motility with effects on satiation. Sci Rep. 2015;5:15985. 26541810 10.1038/srep15985 PMC4635351

[2] Janssen S , Laermans J, Verhulst PJ, Thijs T, Tack J, Depoortere I. Bitter taste receptors and alpha-gustducin regulate the secretion of ghrelin with functional effects on food intake and gastric emptying. Proc Natl Acad Sci USA. 2011;108(5):2094-2099. 21245306 10.1073/pnas.1011508108 PMC3033292

[3] Kim KS , Egan JM, Jang HJ. Denatonium induces secretion of glucagon-like peptide-1 through activation of bitter taste receptor pathways. Diabetologia. 2014;57(10):2117-2125. 25016595 10.1007/s00125-014-3326-5 PMC5160131

[4] Pham H , Hui H, Morvaridi S, et al A bitter pill for type 2 diabetes? The activation of bitter taste receptor TAS2R38 can stimulate GLP-1 release from enteroendocrine L-cells. Biochem Biophys Res Commun. 2016;475(3):295-300. 27208775 10.1016/j.bbrc.2016.04.149 PMC4918516

[5] Rezaie P , Bitarafan V, Horowitz M, Feinle-Bisset C. Effects of bitter substances on GI function, energy intake and glycaemia-so preclinical findings translate to outcomes in humans? Nutrients. 2021;13(4):1317. 33923589 10.3390/nu13041317 PMC8072924

[6] Wu SV , Rozengurt N, Yang M, Young SH, Sinnett-Smith J, Rozengurt E. Expression of bitter taste receptors of the T2R family in the gastrointestinal tract and enteroendocrine STC-1 cells. Proc Natl Acad Sci USA. 2002;99(4):2392-2397. 11854532 10.1073/pnas.042617699 PMC122375

[7] Chen MC , Wu SV, Reeve JR Jr, Rozengurt E. Bitter stimuli induce Ca2+ signaling and CCK release in enteroendocrine STC-1 cells: role of L-type voltage-sensitive Ca2+ channels. Am J Physiol Cell Physiol. 2006;291(4):C726-C739. 16707556 10.1152/ajpcell.00003.2006

[8] Le Nevé B , Foltz M, Daniel H, Gouka R. The steroid glycoside H.g.-12 from Hoodia gordonii activates the human bitter receptor TAS2R14 and induces CCK release from HuTu-80 cells. Am J Physiol Gastrointest Liver Physiol. 2010;299(6):G1368-G1375. 20930049 10.1152/ajpgi.00135.2010

[9] Wang Q , Liszt KI, Deloose E, et al Obesity alters adrenergic and chemosensory signaling pathways that regulate ghrelin secretion in the human gut. FASEB J. 2019;33(4):4907-4920. 30629462 10.1096/fj.201801661RR

[10] Okitolonda W , Delacollette C, Malengreau M, Henquin JC. High incidence of hypoglycaemia in African patients treated with intravenous quinine for severe malaria. Br Med J (Clin Res Ed). 1987;295(6600):716-718. 10.1136/bmj.295.6600.716 PMC1247739 3117315

[11] Henquin JC . Quinine and the stimulus-secretion coupling in pancreatic beta-cells: glucose-like effects on potassium permeability and insulin release. Endocrinol. 1982;110(4):1325-1332. 10.1210/endo-110-4-1325 6277599

[12] Andreozzi P , Sarnelli G, Pesce M, et al The bitter taste receptor agonist quinine reduces calorie intake and increases the postprandial release of cholecystokinin in healthy subjects. J Neurogastroenterol Motil. 2015;21(4):511-519. 26351252 10.5056/jnm15028 PMC4622133

[13] Bitarafan V , Fitzgerald PCE, Little TJ, et al Intragastric administration of the bitter tastant quinine lowers the glycemic response to a nutrient drink without slowing gastric emptying in healthy men. Am J Physiol Regul Integr Comp Physiol. 2020;318(2): R263-R273. 31774306 10.1152/ajpregu.00294.2019

[14] Deloose E , Corsetti M, Van Oudenhove L, Depoortere I, Tack J. Intragastric infusion of the bitter tastant quinine suppresses hormone release and antral motility during the fasting state in healthy female volunteers. J Neurogastroenterol Motil. 2018;30(1):e13171. 10.1111/nmo.13171 28776826

[15] Iven J , Biesiekierski JR, Zhao D, et al Intragastric quinine administration decreases hedonic eating in healthy women through peptide-mediated gut-brain signaling mechanisms. Nutr Neurosci. 2019;22(12):850-862. 29607741 10.1080/1028415X.2018.1457841

[16] Rose BD , Bitarafan V, Rezaie P, Fitzgerald PCE, Horowitz M, Feinle-Bisset C. Comparative effects of intragastric and intraduodenal administration of quinine on the plasma glucose response to a mixed-nutrient drink in healthy men: relations with glucoregulatory hormones and gastric emptying. J Nutr. 2021;151(6):1453-1461. 33704459 10.1093/jn/nxab020

[17] Verbeure W , Deloose E, Tóth J, et al The endocrine effects of bitter tastant administration in the gastrointestinal system: intragastric versus intraduodenal administration. Am J Physiol Endocrinol Metab. 2021;321(1):E1-E10. 34029163 10.1152/ajpendo.00636.2020

[18] Imai H , Hakukawa M, Hayashi M, Iwatsuki K, Masuda K. Expression of bitter taste receptors in the intestinal cells of non-human primates. Int J Mol Sci. 2020;21(3):902. 10.3390/ijms21030902 PMC7037741 32019181

[19] Bartoshuk LM , Duffy VB, Miller IJ. PTC/PROP tasting: anatomy, psychophysics, and sex effects. Physiol Behav. 1994;56(6):1165-1171. 7878086 10.1016/0031-9384(94)90361-1

[20] Deloose E , Janssen P, Corsetti M, et al Intragastric infusion of denatonium benzoate attenuates interdigestive gastric motility and hunger scores in healthy female volunteers. Am J Clin Nutr. 2017;105(3):580-588. 28148502 10.3945/ajcn.116.138297

[21] Steinert RE , Luscombe-Marsh ND, Little TJ, et al Effects of intraduodenal infusion of L-tryptophan on ad libitum eating, antropyloroduodenal motility, glycemia, insulinemia, and gut peptide secretion in healthy men. J Clin Endocrinol Metab. 2014;99(9):3275-3284. 24926954 10.1210/jc.2014-1943

[22] Stunkard AJ , Messick S. The three-factor eating questionnaire to measure dietary restraint, disinhibition and hunger. J Psychosom Res. 1985;29(1):71-83. 3981480 10.1016/0022-3999(85)90010-8

[23] Chalé-Rush A , Burgess JR, Mattes RD. Evidence for human orosensory (taste?) sensitivity to free fatty acids. Chem Senses. 2007;32(5):423-431. 17361006 10.1093/chemse/bjm007

[24] Brennan IM , Feltrin KL, Nair NS, et al Effects of the phases of the menstrual cycle on gastric emptying, glycemia, plasma GLP-1 and insulin, and energy intake in healthy lean women. Am J Physiol Gastrointest Liver Physiol. 2009;297(3):G602-G610. 19556358 10.1152/ajpgi.00051.2009

[25] Steinert RE , Landrock MF, Ullrich SS, et al Effects of intraduodenal infusion of the branched-chain amino acid leucine on ad libitum eating, gut motor and hormone functions, and glycemia in healthy men. Am J Clin Nutr. 2015;102(4):820-827. 26289436 10.3945/ajcn.115.114488

[26] Heddle R , Dent J, Toouli J, Read NW. Topography and measurement of pyloric pressure waves and tone in humans. Am J Physiol. 1988;255(4 Pt 1):G490-G497. 3140675 10.1152/ajpgi.1988.255.4.G490

[27] Rehfeld JF . Accurate measurement of cholecystokinin in plasma. Clin Chem. 1998;44(5):991-1001. 9590372

[28] Pilichiewicz AN , Little TJ, Brennan IM, et al Effects of load, and duration, of duodenal lipid on antropyloroduodenal motility, plasma CCK and PYY, and energy intake in healthy men. Am J Physiol Regul Integr Comp Physiol. 2006;290(3):R668-R677. 16210415 10.1152/ajpregu.00606.2005

[29] Feltrin KL , Little TJ, Meyer JH, et al Effects of lauric acid on upper gut motility, plasma cholecystokinin and peptide YY, and energy intake are load, but not concentration, dependent in humans. J Physiol. 2007;581(Pt 2):767-777. 17331985 10.1113/jphysiol.2007.129650 PMC2075194

[30] Heddle R , Dent J, Read NW, et al Antropyloroduodenal motor responses to intraduodenal lipid infusion in healthy volunteers. Am J Physiol. 1988;254(5 Pt 1):G671-G679. 3364568 10.1152/ajpgi.1988.254.5.G671

[31] Camilleri M , Malagelada JR. Abnormal intestinal motility in diabetics with the gastroparesis syndrome. Eur J Clin Invest. 1984;14(6):420-427. 6441717 10.1111/j.1365-2362.1984.tb01206.x

[32] Feltrin KL , Patterson M, Ghatei MA, et al Effect of fatty acid chain length on suppression of ghrelin and stimulation of PYY, GLP-2 and PP secretion in healthy men. Peptides. 2006;27(7):1638-1643. 16563563 10.1016/j.peptides.2006.01.023

[33] McVeay C , Fitzgerald PCE, Ullrich SS, Steinert RE, Horowitz M, Feinle-Bisset C. Effects of intraduodenal administration of lauric acid and L-tryptophan, alone and combined, on gut hormones, pyloric pressures, and energy intake in healthy men. Am J Clin Nutr. 2019;109(5):1335-1343. 31051504 10.1093/ajcn/nqz020

[34] Ryan AT , Luscombe-Marsh ND, Saies AA, et al Effects of intraduodenal lipid and protein on gut motility and hormone release, glycemia, appetite, and energy intake in lean men. Am J Clin Nutr. 2013;98(2):300-311. 23803895 10.3945/ajcn.113.061333

[35] Hogben CA , Tocco DJ, Brodie BB, Schanker LS. On the mechanism of intestinal absorption of drugs. J Pharmacol Exp Ther. 1959;125(4):275-282. 13642268

[36] Murakami M , Takada K, Muranishi S. Further mechanistic study on intestinal absorption enhanced by unsaturated fatty acids: reversible effect by sulfhydryl modification. Biochim Biophys Acta. 1992;1117(1):83-89. 1627597 10.1016/0304-4165(92)90166-r

[37] Lin HC , Doty JE, Reedy TJ, Meyer JH. Inhibition of gastric emptying by glucose depends on length of intestine exposed to nutrient. Am J Physiol. 1989;256(2 Pt 1):G404-G411. 2919683 10.1152/ajpgi.1989.256.2.G404

[38] Little TJ , Doran S, Meyer JH, et al The release of GLP-1 and ghrelin, but not GIP and CCK, by glucose is dependent upon the length of small intestine exposed. Am J Physiol Endocrinol Metab. 2006;291(3):E647-E655. 16684852 10.1152/ajpendo.00099.2006

[39] Meyer JH , Hlinka M, Tabrizi Y, DiMaso N, Raybould HE. Chemical specificities and intestinal distributions of nutrient-driven satiety. Am J Physiol. 1998;275(4):R1293-R1307. 9756563 10.1152/ajpregu.1998.275.4.R1293

[40] Gribble FM , Reimann F. Function and mechanisms of enteroendocrine cells and gut hormones in metabolism. Nat Rev Endocrinol. 2019;15(4):226-237. 30760847 10.1038/s41574-019-0168-8

[41] Steinert RE , Feinle-Bisset C, Asarian L, Horowitz M, Beglinger C, Geary N. Ghrelin, CCK, GLP-1, and PYY(3-36): secretory controls and physiological roles in eating and glycemia in health, obesity, and after RYGB. Physiol Rev. 2017;97(1):411-463. 28003328 10.1152/physrev.00031.2014 PMC6151490

[42] Little TJ , Feltrin KL, Horowitz M, et al Dose-related effects of lauric acid on antropyloroduodenal motility, gastrointestinal hormone release, appetite, and energy intake in healthy men. Am J Physiol Regul Integr Comp Physiol. 2005;289(4):R1090-R1098. 15961531 10.1152/ajpregu.00290.2005

[43] Parker BA , Doran S, Wishart J, Horowitz M, Chapman IM. Effects of small intestinal and gastric glucose administration on the suppression of plasma ghrelin concentrations in healthy older men and women. Clin Endocrinol (Oxf). 2005;62(5):539-546. 15853822 10.1111/j.1365-2265.2005.02254.x

[44] Batterham RL , Cohen MA, Ellis SM, et al Inhibition of food intake in obese subjects by peptide YY3-36. N Engl J Med. 2003;349(10):941-948. 12954742 10.1056/NEJMoa030204

[45] Brennan IM , Otto B, Feltrin KL, Meyer JH, Horowitz M, Feinle-Bisset C. Intravenous CCK-8, but not GLP-1, suppresses ghrelin and stimulates PYY release in healthy men. Peptides. 2007;28(3):607-611. 17129639 10.1016/j.peptides.2006.10.014

[46] Degen L , Oesch S, Casanova M, et al Effect of peptide YY3-36 on food intake in humans. Gastroenterology. 2005;129(5):1430-1436. 16285944 10.1053/j.gastro.2005.09.001

[47] Nauck MA , Heimesaat MM, Behle K, et al Effects of glucagon-like peptide-1 on counterregulatory hormone responses, cognitive functions, and insulin secretion during hyperinsulinemic, stepped hypoglycemic clamp experiments in healthy volunteers. J Clin Endocrinol Metab. 2002;87(3):1239-1246. 11889194 10.1210/jcem.87.3.8355

[48] Henquin JC , Horemans B, Nenquin M, Verniers J, Lambert AE. Quinine-induced modifications of insulin release and glucose metabolism by isolated pancreatic islets. FEBS Lett. 1975;57(3):280-284. 1102333 10.1016/0014-5793(75)80317-6

[49] Houghton LA , Read NW, Heddle R, et al Relationship of the motor activity of the antrum, pylorus, and duodenum to gastric emptying of a solid-liquid mixed meal. Gastroenterology. 1988;94(6):1285-1291. 3360256 10.1016/0016-5085(88)90665-8

[50] Smith GT , Moran TH, Coyle JT, Kuhar MJ, O’Donahue TL, McHugh PR. Anatomic localization of cholecystokinin receptors to the pyloric sphincter. Am J Physiol Regul Integr Comp Physiol. 1984;246(1):R127-R130. 10.1152/ajpregu.1984.246.1.R127 6320669

[51] Garneau NL , Nuessle TM, Sloan MM, Santorico SA, Coughlin BC, Hayes JE. Crowdsourcing taste research: genetic and phenotypic predictors of bitter taste perception as a model. Front Integr Neurosci. 2014;8:33. 24904324 10.3389/fnint.2014.00033 PMC4035552

[52] Stewart JE , Seimon RV, Otto B, Keast RS, Clifton PM, Feinle-Bisset C. Marked differences in gustatory and gastrointestinal sensitivity to oleic acid between lean and obese men. Am J Clin Nutr. 2011;93(4):703-711. 21310831 10.3945/ajcn.110.007583

[53] Horie I , Abiru N, Eto M, et al Sex differences in insulin and glucagon responses for glucose homeostasis in young healthy Japanese adults. J Diabetes Investig. 2018;9(6):1283-1287. 10.1111/jdi.12829 PMC6215950 29489067

